# Inhibition of HDAC3 Expression Alleviates High–Glucose‐Induced Photoreceptor Cell Apoptosis and Oxidative Stress

**DOI:** 10.1155/jdr/5747601

**Published:** 2026-04-30

**Authors:** Qi Fang, Jiali Li, Baoyi Liu, Yulin Ma, Jiaqi Liu, Qiuxia Lin, Lin Ling, Songfu Feng

**Affiliations:** ^1^ Hangzhou Linping District Maternal & Child Health Care Hospital, Hangzhou, China; ^2^ Department of Ophthalmology, Zhujiang Hospital, Southern Medical University, Guangzhou, China, fimmu.com; ^3^ Department of Ophthalmology, GuangZhou Red Cross Hospital of Jinan University, Guangzhou, China, jnu.edu.cn

**Keywords:** apoptosis, diabetic retinopathy, histone deacetylase 3, oxidative stress, photoreceptor cell

## Abstract

**Background:**

Diabetic retinopathy (DR), the most common ocular complication of diabetes mellitus, is a leading cause of blindness among the working‐age population. Its pathogenesis is mainly related to blood–retinal barrier destruction, inflammation, retinal neuron damage, oxidative stress, and genetic immune factors. This study is aimed at elucidating the role and underlying mechanism of histone deacetylase 3 (HDAC3) in DR‐associated retinal neuronal injury.

**Methods:**

In this study, the mouse model of diabetes was induced by intraperitoneal injection of streptozotocin (STZ) dissolved in sodium citrate buffer. At 4, 8, and 12 weeks, the thickness of the retinal and photoreceptor layers was observed by hematoxylin–eosin staining, and the expression of HDAC3 in the mouse retina was evaluated by immunohistochemical staining. In vitro, 661W cells were cultured under high‐glucose conditions to mimic the diabetic environment. To investigate the role of HDAC3, its expression was inhibited using either the specific inhibitor RGFP966 or HDAC3‐specific small interfering RNA (HDAC3‐siRNA). Subsequently, the levels of oxidative stress and apoptosis were detected to analyze the mechanism by which HDAC3 influences photoreceptor damage.

**Results:**

With the prolongation of diabetes duration, the thickness of the retinal layer and photoreceptor cell layer becomes thinner, and the expression of HDAC3 in the retina increases. In vitro, HDAC3 expression, apoptosis, and oxidative stress were increased in 661W cells treated with high glucose. Critically, inhibition of HDAC3 using either RGFP966 or HDAC3‐siRNA effectively attenuated the high–glucose‐induced apoptosis and oxidative stress in these cells.

**Conclusion:**

Our results suggest that HDAC3 is associated with apoptosis and oxidative stress of DR photoreceptors, and the inhibition of HDAC3 can reduce apoptosis and oxidative stress of DR photoreceptors. It is suggested that epigenetic therapy of HDAC3 inhibitors may have therapeutic value in the prevention and treatment of DR.

## 1. Introduction

Diabetic retinopathy (DR) is one of the most prevalent neurological and microvascular complications of diabetes mellitus (DM) and represents the leading cause of visual impairment and blindness among working‐age adults globally [1, 2]. Over the past few decades, the worldwide prevalence of diabetes has escalated due to various factors, including socioeconomic development and lifestyle changes [3–5]. DR is a multifactorial disease with complex pathogenesis [6]. While traditionally viewed as a microvascular disorder characterized by abnormalities in the retinal vasculature [7], its pathological features also include increased blood–retinal barrier permeability, loss of pericytes and endothelial cells, basement membrane thickening, capillary nonperfusion, and neovascularization secondary to retinal ischemia and hypoxia [8, 9]. Historically, research on DR has predominantly focused on vascular abnormalities. Recent studies have provided increasing evidence that DR is not merely a microvascular disease but also involves retinal neurodegenerative changes [10]. Moreover, retinal neurodegeneration may occur even before detectable microvascular damage [11–14]. Clinical manifestations of DR‐related neurodegeneration include delayed dark adaptation, reduced contrast sensitivity, and impaired tonal discrimination [15]. Photoreceptor cells, as the most abundant and metabolically active special neuron cells, are crucial for light signal transduction and visual function imaging [16, 17]. At present, studies have suggested that the severity of DR and visual impairment are related to changes in the photoreceptor layer [18], including morphological shrinkage, dysfunction and apoptosis of photoreceptor cells [19]. Studies utilizing optical coherence tomography (OCT) and optical coherence tomography angiography (OCTA) have shown that the neurovascular units of diabetic patients without DR have been damaged [20].

Histone deacetylases (HDACs) are a class of enzymes that modify chromatin structure and play critical roles in regulating gene expression, cell proliferation, apoptosis, migration, differentiation, oxidative stress, and inflammation [21, 22]. Mammals possess four classes of HDACs, and substantial evidence implicates their involvement in the pathogenesis of diabetes and its complications [23]. In particular, HDAC3 has been recently linked to various diabetic complications, including diabetic liver disease, aortic atherosclerosis, osteoporosis, cardiomyopathy, and nephropathy [24–27]. At the same time, HDAC3 also plays an important role in ocular diseases, such as DR and optic nerve injury. In the model of acute optic nerve injury, the expression of HDAC3 is associated with apoptosis of retinal ganglion cells (RGCs), and inhibition of HDAC3 expression has neuroprotective effects [28]. Elevated HDAC3 expression has been observed in the retinal tissues of diabetic mice. Inhibition of HDAC3 expression has been demonstrated to alleviate DR progression and reduce apoptosis and oxidative stress activated by DR [29]. Research found that HDAC3 expression was increased in RGCs of db/db mouse and positively correlated with cellular apoptosis and autophagy [30]. These findings indicate that HDAC3 is an important factor influencing oxidative stress and apoptosis of retinal cells in DR. However, the specific pathological changes in photoreceptors during DR progression, and whether and how HDAC3 regulates photoreceptor pathophysiology, remain largely unexplored.

In this study, we aim to investigate the role and mechanism of HDAC3 in retinal neurodegeneration associated with DR. Utilizing a diabetic mouse model and 661W photoreceptor cells cultured under high glucose conditions, we explore the impact of HDAC3 expression on oxidative stress and apoptosis. By inhibiting HDAC3 through selective inhibitors and siRNA, we seek to determine its potential as a therapeutic target. Our findings may provide new insights into epigenetic‐based strategies for the prevention and treatment of DR.

## 2. Methods

### 2.1. Construction of Diabetic Mouse Model

The same batch of SPF C57BL/6 mice without eye disease and endocrine disease was selected and randomly divided into control group and DM group. All the mice were acclimatized in the animal center for 1 week before proceeding with the subsequent operations. The control group was fed a standard maintenance diet. The DM group was intraperitoneally injected with streptozotocin (STZ) sodium citrate solution (dose: 0.05 mL/10 g body weight) and was maintained on a high‐sugar diet (66.5% standard maintenance diet + 10% lard + 20% sucrose + 2.5% cholesterol + 1% sodium cholate). All mice were placed in controlled environmental conditions (temperature: 21°C; humidity: 60%; 12‐h light/dark cycle) with ad libitum access to food and water. Body weight and blood glucose levels (measured via tail vein sampling) were monitored regularly. All animal experiments in this study were approved by the Medical Ethics Committee of Zhujiang Hospital of Southern Medical University (LAEC‐2023‐172) and followed the guidelines of the Helsinki Declaration.

### 2.2. Hematoxylin–Eosin (HE) Staining of Mouse Retina

Retinal tissues were randomly harvested from mice in each group. The specimens were fixed, embedded in paraffin, and sectioned at a thickness of 4 *μ*m. Following deparaffinization and rehydration, the sections were stained with HE using standard protocols to evaluate histopathological morphology under a light microscope. The images of three sections with the same distance from the optic nerve in each region were analyzed to quantify the total thickness of the retina and the thickness of the retinal photosensitive cell layer.

### 2.3. Immunohistochemical Staining of Mouse Retina

Retinal tissues were randomly selected from mice in each group. Paraffin‐embedded tissues were sectioned at a thickness of 4 *μ*m. Following deparaffinization and hydration, antigen retrieval was performed by microwave heating in citrate buffer for 20 min. The sections were cleaned with PBS for three times. Subsequently, sections were incubated overnight with HDAC3 antibody (1:200) at 4°C. After that, the eye sections were washed with PBS for three times (5 min per wash). Immunoreactivity was visualized using a DAB substrate kit according to the manufacturer′s instructions, with the development time carefully monitored under a microscope. The reaction was stopped by rinsing with distilled water. Finally, the sections were counterstained with hematoxylin for 3 min, followed by washing, dehydration, clearing in xylene, and mounting with a coverslip. Stained sections were observed under a light microscope. The expression of HDAC3 in the retina was observed by image analysis of three sections with the same distance of optic nerve in each region.

### 2.4. Cell Culture and Treatment

The 661W cell line was purchased from Wuxi Xin Run Biotechnology Co. Ltd and passed the test of short tandem repeats (STR). The 661W was maintained in Dulbecco′s modified Eagle′s medium (DMEM) containing 4.5 g/L (24.5 mM) glucose, and supplemented with 10% heat‐inactivated fetal bovine serum (FBS) and 1% penicillin–streptomycin solution. For the experiments, 661W cells were maintained in 24.5 mM glucose (control group) or cultured in 55 mM glucose (HG group), and both groups were incubated in a cell incubator at 37°C, 5% CO_2_, and 70%–80% humidity. The experiment was carried out after the cell culture reached 80%–90% fusion degree.

### 2.5. Cell Counting Kit‐8 (CCK‐8) Assay

Cell viability of 661W cells was assessed using a CCK‐8. Briefly, cells were seeded into 96‐well plates at a density of 5 × 10^3^cells per well in 100 *μ*L of culture medium, with five replicate wells per experimental group. After treatment for 48 h, 90‐*μ*L culture solution and 10‐*μ*L CCK‐8 solution were added, respectively, and cultured at 37°C for 2 h. The optical density at 450 nm was measured by enzyme‐labeled instrument, and the data were recorded and analyzed.

### 2.6. Real‐Time PCR

Total RNA was isolated from 661W cells using TRIzol reagent. cDNA was reverse transcribed by superscript first strand synthesis system. Then, quantitative PCR was performed using Power SYBRGreen PCR MasterMix. The primer sequences were shown as follows:HDAC3 (Fwd: 5 ^′^‐GGCGACCATGACAACGACAAG; Rev: 5 ^′^‐CATACTTTCCTTCCCACCACAGAG),*β*‐actin (Fwd: 5 ^′^‐TGGCACCACACCTTCTACAATGAG; Rev: 5 ^′^‐GAGGCATACAGGGACAGCACAG),Bcl‐2 (Fwd:5 ^′^‐CCAGAGGTCTCAGAGAACAGGATG;Rev: 5 ^′^‐TCTTGGCACCAYAGCAGCACAG); Bax (Fwd: 5 ^′^‐ATGGGCTGGACACTGGA;Rev: 5 ^′^‐GGTGAGCGAGGCGGTGAG),Caspase‐3 (Fwd:5 ^′^‐GGCGACTTCCTGTATGCTTACTC; Rev: 5 ^′^‐CGACCCGTCCTTTGAATTTCTCC).

### 2.7. Western Blotting

Total protein was extracted from photoreceptor cells and mouse retinal tissues. The protein concentration was quantified using a Pierce BCA protein assay kit (KeyGEN, China). Equal amounts of proteins (20 *μ*g per lane) were separated by sodium dodecyl sulfate–polyacrylamide gel electrophoresis (SDS‐PAGE) and transferred to polyvinylidene difluoride (PVDF) membranes (Merck Millipore). After electro‐blotting onto PVDF membranes, the membranes were blocked with 5% BSA for 1 h at room temperature. Then the membranes were incubated at 4°C overnight with the following primary antibodies: HDAC3 (1:1000, Affinity, United States), glyceraldehyde 3‐phosphate dehydrogenase (GAPDH) (1:1000, Fude Biotechnology, Hangzhou, China), Bcl‐2 (1:1000, Aibotaike Biotechnology, Wuhan, China), Bax (1:1000, Abmart, Shanghai, China), and Caspase3 (1:1000, Aibotaike Biotechnology, Wuhan, China). The membranes were washed three times with 0.1% TBST and incubated with secondary antibodies for 1 h at room temperature. Last, the protein bands were visualized using an ECL‐plus western blotting detection system. Analysis of each protein band was performed using ImageJ software. The expression of GAPDH was used as an internal control.

### 2.8. Preparation of RGFP966 Solution

RGFP966, a highly selective HDAC3 inhibitor, was dissolved in dimethyl sulfoxide (DMSO). A 15‐mM stock solution was prepared by adding 5 mg of RGFP966 to 1.37 mL of DMSO, followed by ultrasonic agitation to ensure complete dissolution. The solution was aliquoted and stored at −80°C. Prior to use, an aliquot was thawed at 4°C and diluted into cell culture medium to the desired working concentration. To account for potential effects of the solvent, an additional control group (HG + DMSO) was included in the experiments, in which cells were exposed to high glucose medium containing an equivalent volume of DMSO.

### 2.9. Transfection With siRNA

The 661W cells were seeded in culture plates for 24 h. Transient transfection was performed using HDAC3‐specific siRNA (stock concentration: 50 *μ*M). For each transfection, 4 *μ*L of siRNA was diluted in 200 *μ*L of jetPRIME buffer, mixed with 4 *μ*L of jetPRIME transfection reagent (Polyplus‐transfection S.A., Illkirch, France), and incubated according to the manufacturer′s instructions. The mixture was then added to the cells. After 48 h of transfection, cells were harvested for subsequent experiments.

### 2.10. Measurement of Malondialdehyde (MDA) Content

The MDA content in 661W cells was measured using a commercial MDA assay kit. Cells were lysed by ultrasonication according to the manufacturer′s instructions. The lysates were then used to determine total MDA content. Absorbance was measured at 532 and 600 nm using a multifunctional microplate reader, and the MDA concentration was calculated based on the difference in absorbance at these wavelengths.

### 2.11. Measurement of Total Superoxide Dismutase (SOD) Activity

SOD activity in 661W cells was determined using a commercial SOD assay kit. Cells were lysed by ultrasonication following the manufacturer′s protocol. The resulting lysates were used to measure SOD activity by detecting absorbance at 450 nm with a multifunctional microplate reader. Data were recorded and analyzed.

### 2.12. Detection of Reactive Oxygen Species (ROS)

The 661W cells in the logarithmic growth stage were seeded onto glass coverslips. The intracellular ROS levels were measured using a ROS assay kit based on dihydroethidium (DHE), which permeates live cells and is oxidized by ROS to produce red fluorescence. After staining, a drop of DAPI‐free antifluorescence attenuation sealer was applied to a glass slide, and the coverslip with cells was placed facedown onto the medium. The mounted samples were kept in a dark humidified chamber before imaging. The images were immediately collected and analyzed using a Nikon inverted microscope.

### 2.13. Assessment of Apoptosis by Flow Cytometry

Apoptosis was evaluated using an Annexin V‐FITC/PI apoptosis detection kit. A total of 5 *μ*L Annexin V‐FITC Reagent and 5 *μ*L PI reagent (50 *μ*g/mL) were added to 661W cell suspension according to kit instructions. After gentle swirl mixing, the cells were incubated at room temperature in the dark for 15–20 min. Flow cytometry was used immediately (FITC channel for AnnexinV‐FITC and PerCP channel for PI). Record the data and analyze it.

### 2.14. Statistical Analysis

All experiments were independently repeated there times. The results were statistically analyzed and plotted using GraphPad Prism 8 (Graphpad, San Diego, California, United States). The data were expressed as the mean ± SD. Student′s *t*‐test or one‐way analysis of variance (ANOVA) was used for comparing the differences between groups. Statistically significant was accepted at ∗*p* < 0.05, ∗∗*p* < 0.01, ∗∗∗*p* < 0.001, and ∗∗∗∗*p* < 0.0001.

## 3. Results

### 3.1. The Thickness of the Whole Retinal Layer and Photoreceptor Cell Layer Changed in Diabetic Mice

**Figure 1:**
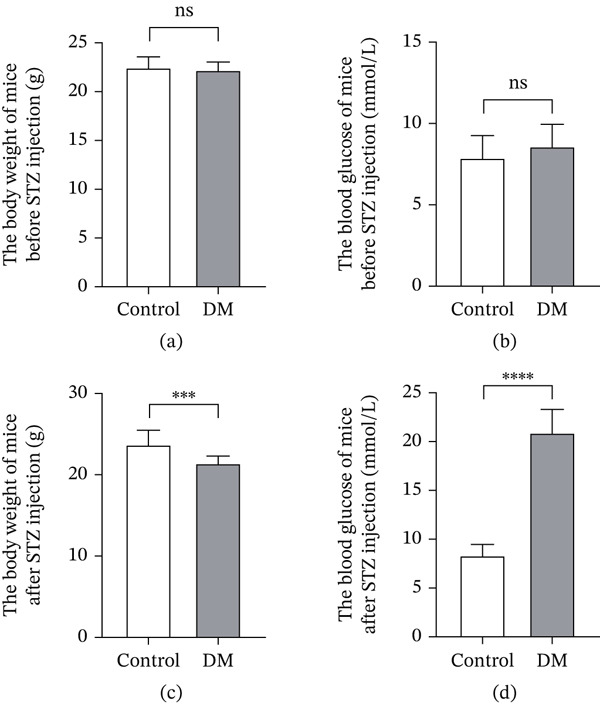
Body weight and random blood glucose levels before and after model induction. (a) The weight of mice in the control and DM groups before modeling. (b) The random blood glucose of mice in the control and DM groups before modeling. (c) The weight of mice in the control and DM groups 2 weeks after modeling. (d) The random blood glucose of mice in the control and DM groups 2 weeks after modeling. Control *n* = 12, DM *n* = 12. ns, not significant, ∗*p* < 0.05, ∗∗*p* < 0.01, ∗∗∗*p* < 0.001, and ∗∗∗∗*p* < 0.0001.

Prior to STZ administration, no significant differences in body weight or blood glucose levels were observed between the control and DM groups (Figure 1a,b). Following five consecutive days of intraperitoneal STZ injections and 2 weeks of a high‐sugar diet, the DM group exhibited significantly lower mean body weight and markedly elevated random blood glucose levels compared with the control group, exceeding the diagnostic threshold for diabetes (> 16.7 mmol/L) (Figure 1c,d). DM mice also displayed characteristic polyphagia, polydipsia, and polyuria. These findings collectively confirmed the successful establishment of the diabetic mouse model.

**Figure 2:**
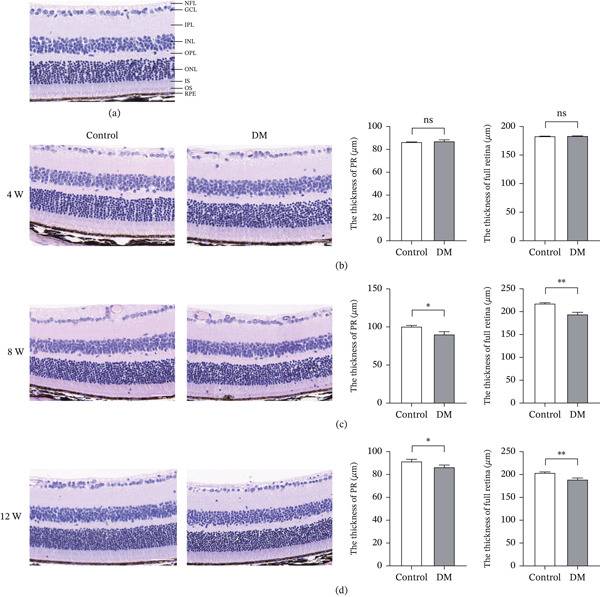
The thickness of the retinal photoreceptor layer became thinner in DM mice. (a) Hematoxylin–eosin (HE) staining of mouse retina paraffin section. NFL is the nerve fiber layer, GCL is the ganglion cell layer, IPL is the inner plexiform layer, INL is the inner nuclear layer, OPL is the outer plexiform layer, ONL is the outer nuclear layer, IS is the inner segment of photoreceptors, OS is the outer segment of photoreceptors, and RPE is the retinal pigment epithelium. (b) HE staining of retinal paraffin sections of the control and DM groups mice at 4 weeks after modeling, and comparison of full retina thickness and PR layer thickness, control *n* = 4, DM *n* = 4. (c) HE staining of retinal paraffin sections of the control and DM groups mice at 8 weeks after modeling, and comparison of full retina thickness and PR layer thickness, control *n* = 4, DM *n* = 4. (d) HE staining of retinal paraffin sections of the control and DM groups mice at 12 weeks after modeling, and comparison of full retina thickness and PR layer thickness, control *n* = 4, DM *n* = 4. Scale = 100 *μ*m. *n* = 3, ns, not significant, ∗*p* < 0.05, ∗∗*p* < 0.01, ∗∗∗*p* < 0.001, and ∗∗∗∗*p* < 0.0001.

In order to compare the pathological changes of retinal structure in STZ‐induced diabetic mice, HE staining was performed on retinal sections from control and DM mice at 4, 8, and 12 weeks postmodeling. The schematic diagram of the various layers of the mouse retina is shown in the figure (Figure 2a). We measured the thickness of the full retina layer and the PR layer in mice, where the PR layer encompasses the IS, OS, and ONL layers. The results of HE staining showed that there was no significant difference in the thickness of the full retina layer and PR layer between the control group and DM group at 4 weeks. The tissue structure of each layer of the retina was clear and the cells were arranged tightly (Figure 2b). By 8 weeks, the DM group showed a noticeable reduction in both full retinal thickness and PR layer thickness compared with the control group (Figure 2c). At the 12‐week time point, retinal thinning progressed further in DM mice, accompanied by aggravated pathological features including disorganized laminar structure, loss of distinct layer boundaries, and loosened cellular arrangement in the inner and outer nuclear layers (Figure 2d).

### 3.2. HDAC3 Expression is Upregulated in the Retina of Diabetic Mice

**Figure 3:**
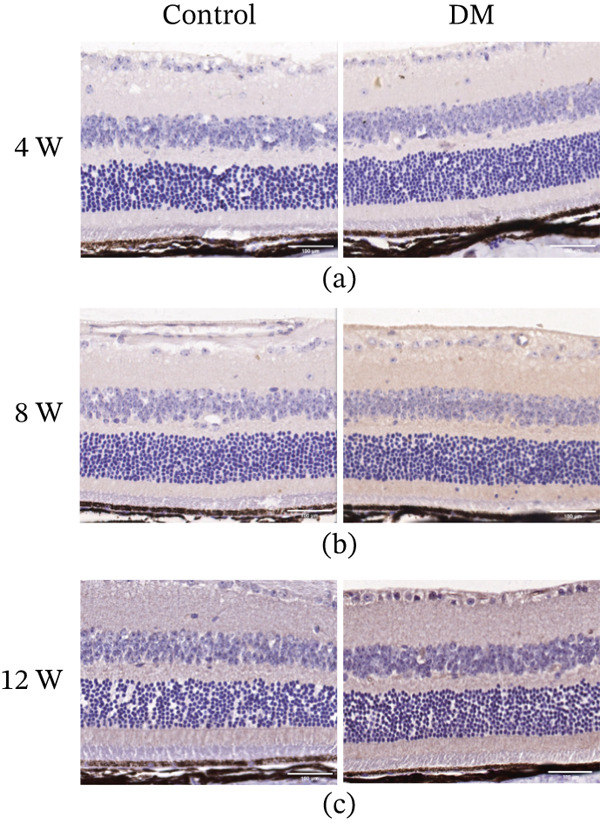
HDAC3 was upregulated in the retina of DM mice. Immunohistochemical staining of retinal paraffin sections of the control and DM groups mice at (a) 4 weeks, (b) 8 weeks, and (c) 12 weeks after modeling. Scale = 100 *μ*m. Control *n* = 4, DM *n* = 4.

Immunohistochemical analysis revealed no marked difference in HDAC3 expression in the retina between the control and DM groups at 4 weeks after modeling (Figure 3a). However, at 8 and 12 weeks postmodeling, HDAC3 expression was increased in the DM group compared with the control group, as indicated by intensified immunostaining (Figure 3b,c). The above results demonstrate that HDAC3 expression in the retina rises with the progression of diabetes.

### 3.3. HDAC3 Expression Is Upregulated in 661W Cells Under High Glucose Conditions

**Figure 4:**
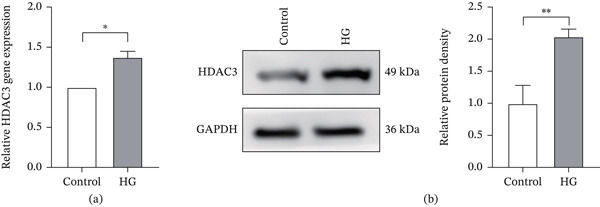
HDAC3 was upregulated in 661W cells under high glucose environment. (a) Detection of the relative expression of HDAC3 mRNA in the 661W cells of the control and HG groups by qPCR. (b) Detection of the relative protein expression of HDAC3 in the 661W cells of the control and HG groups by western blotting. The internal reference is GAPDH, and the bar graph was drawn by ImageJ software to quantify the relative expression of HDAC3 protein in three independent experiments. *n* = 3, ns, not significant, ∗*p* < 0.05, ∗∗*p* < 0.01, ∗∗∗*p* < 0.001, and ∗∗∗∗*p* < 0.0001.

To evaluate HDAC3 expression in photoreceptor cells under hyperglycemic, 661W photoreceptor cells were divided into control group and HG group. Cells in the HG group were cultured in high glucose medium for 48 h to mimic the DR microenvironment in vitro. qRT‐PCR analysis revealed a significant increase in HDAC3 mRNA expression in the HG group compared with the control group (Figure 4a). Consistent with this, western blot analysis of cellular protein extracts showed that HDAC3 protein expression was also markedly upregulated following 48 h of high glucose treatment (Figure 4b).

### 3.4. High Glucose Conditions Promote Apoptosis in 661W Cells

**Figure 5:**
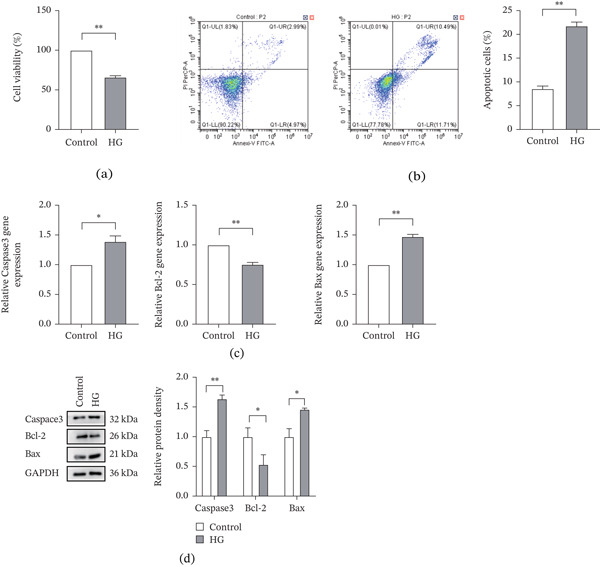
The level of apoptosis increased in 661W under high glucose environment. (a) The cell viability of 661W cells in the control and HG groups detected by CCK8 assay. (b) Detection of the apoptosis level of 661W cells in the control and HG groups by flow cytometry. The total apoptosis rate of each group was counted, and the bar chart was drawn. (c) Detection of the relative expression of Caspase3, Bcl‐2, and Bax mRNA in the 661W cells of the control and HG groups by qPCR. (d) Detection of the relative protein expression of Caspase3, Bcl‐2, and Bax in the 661W cells of the control and HG groups by western blotting. The internal reference is GAPDH, and the bar graph was drawn by ImageJ software to quantify the relative expression of Caspase3, Bcl‐2, and Bax protein in three independent experiments. *n* = 3, ns, not significant, ∗*p* < 0.05, ∗∗*p* < 0.01, ∗∗∗*p* < 0.001, and ∗∗∗∗*p* < 0.0001.

Apoptosis of retinal cells is a key mechanism in the occurrence and development of DR. First of all, we assessed viability of 661W cells in the control and HG group at different time points using the CCK‐8 assay. Results showed that cell viability was significantly reduced in the HG group after 48 h of high glucose stimulation compared with the control group (Figure 5a). Based on this finding, the 48‐h treatment period was selected for subsequent experiments.

The results of flow cytometry revealed that the total apoptosis rate was significantly higher in the HG group than in the control group. Specifically, both early and late apoptosis rates were increased under high glucose conditions (Figure 5b).

We further examined the expression of Caspase‐3, Bax, Bcl‐2, which are important factors in the signal transduction pathway of apoptosis. qRT‐PCR results indicated that after 48 h of high glucose exposure, mRNA expression of Caspase‐3 and Bax was significantly upregulated, whereas Bcl‐2 mRNA was downregulated in the HG group compared with the control group (Figure 5c). Consistent with these findings, western blot analysis confirmed that protein levels of Caspase‐3 and Bax were markedly increased, whereas Bcl‐2 protein expression was significantly decreased in the HG group (Figure 5d).

### 3.5. High Glucose Conditions Enhance Oxidative Stress in 661W Cells

**Figure 6:**
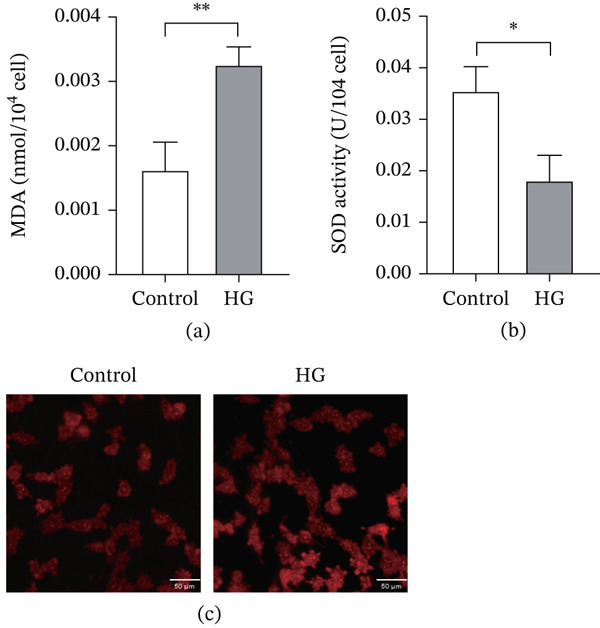
High glucose environment increased the level of apoptosis in 661W. (a) The malondialdehyde (MDA) content of 661W cells in the control and HG groups. (b) The superoxide dismutase (SOD) activity of 661W cells in the control and HG groups. (c) The fluorescence intensity of ROS in 661W cells in the control and HG groups. *n* = 3, ns, not significant, ∗*p* < 0.05, ∗∗*p* < 0.01, ∗∗∗*p* < 0.001, and ∗∗∗∗*p* < 0.0001. Scale = 50 *μ*m.

Oxidative stress plays a key role in the course of DR and promotes the progress of the disease. MDA, SOD, and ROS are important indicators to reflect the degree of intracellular oxidative stress. To evaluate the extent of oxidative stress in 661W cells under high glucose conditions, we measured the levels of MDA, SOD activity, and ROS. After 48 h of high glucose exposure, MDA content was significantly elevated in the HG group compared with the control group (Figure 6a), whereas total SOD activity was markedly reduced (Figure 6b). Concurrently, ROS fluorescence staining revealed stronger red fluorescence intensity in the HG group, indicating higher intracellular ROS levels relative to the control (Figure 6c). These results collectively demonstrate that high glucose treatment induces significant oxidative stress in 661W cells.

### 3.6. RGFP966 Attenuates Apoptosis in 661W Cells Under High Glucose Conditions

**Figure 7:**
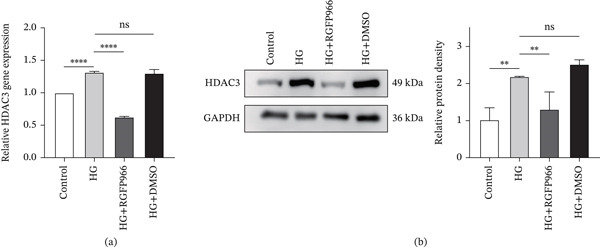
The expression level of HDAC3 in 661W cells was successfully downregulated by RGFP966. (a) Detection of the relative expression of HDAC3 mRNA in the 661W cells of the control, HG, HG + RGFP966, and HG + DMSO groups by qPCR. (b) Detection of the relative protein expression of HDAC3 in the 661W cells of the control, HG, HG + RGFP966, and HG + DMSO groups by western blotting. The internal reference is GAPDH, and the bar graph was drawn by ImageJ software to quantify the relative expression of HDAC3 protein in three independent experiments. *n* = 3, ns, not significant, ∗*p* < 0.05, ∗∗*p* < 0.01, ∗∗∗*p* < 0.001, and ∗∗∗∗*p* < 0.0001.

RGFP966 is a highly selective HDAC3 inhibitor, which can effectively and selectively inhibit the expression of HDAC3. To examine the role of HDAC3 in high–glucose‐mediated apoptosis, 661W cells were divided into four groups: control group, HG group, HG + RGFP966 group, and HG + DMSO group. qRT‐PCR and western blot analyses confirmed that HDAC3 expression was effectively downregulated in the HG + RGFP966 group (Figure 7a,b).

**Figure 8:**
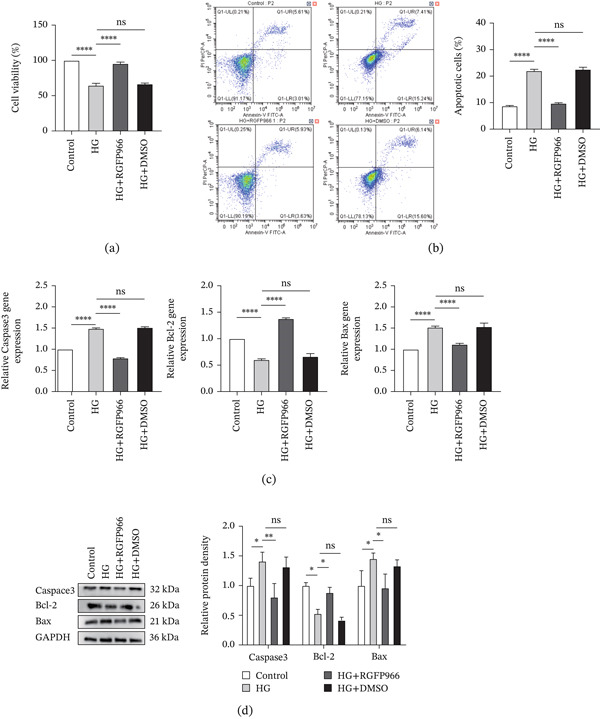
Inhibition of HDAC3 activity reduced the level of apoptosis in 661W. (a) The cell viability of 661W in the control, HG, HG + RGFP966, and HG + DMSO groups detected by CCK8 assay. (b) Detection of the apoptosis level of 661W cells in the control, HG, HG + RGFP966, and HG + DMSO groups by flow cytometry. The total apoptosis rate of each group was counted, and the bar chart was drawn. (c) Detection of the relative expression of Caspase3, Bcl‐2, and Bax mRNA in the 661W cells of the control, HG, HG + RGFP966, and HG + DMSO groups by qPCR. (d) Detection of the relative protein expression of Caspase3, Bcl‐2, and Bax in the 661W cells of the control, HG, HG + RGFP966, and HG + DMSO groups by western blotting. The internal reference is GAPDH, and the bar graph was drawn by ImageJ software to quantify the relative expression of Caspase3, Bcl‐2, and Bax protein in three independent experiments. *n* = 3. ns, not significant, ∗*p* < 0.05, ∗∗*p* < 0.01, ∗∗∗*p* < 0.001, and ∗∗∗∗*p* < 0.0001.

CCK‐8 assay revealed that cell viability was significantly decreased in the HG and HG + DMSO groups compared with the control group after 48 h, whereas supplementation with RGFP966 significantly restored cell viability in the HG + RGFP966 group (Figure 8a). Flow cytometry results indicated that the total apoptosis rate, along with early and late apoptosis rates, was markedly elevated in the HG and HG + DMSO groups relative to the control. In contrast, HDAC3 inhibition significantly reduced all apoptosis indices in the HG + RGFP966 group (Figure 8b).

The results of qRT‐PCR assay showed that high glucose exposure significantly upregulated Caspase‐3 and Bax mRNA expression and downregulated Bcl‐2 expression in the HG and HG + DMSO groups. These effects were alleviated by RGFP966, as evidenced by decreased Caspase‐3 and Bax mRNA levels and increased Bcl‐2 expression in the HG + RGFP966 group (Figure 8c). Western blot results further confirmed these trends at the protein level (Figure 8d). Collectively, these results demonstrate that inhibition of HDAC3 with RGFP966 alleviates high–glucose‐induced apoptosis in 661W cells.

### 3.7. RGFP966 Attenuates Oxidative Stress in 661W Cells Under High Glucose Conditions

**Figure 9:**
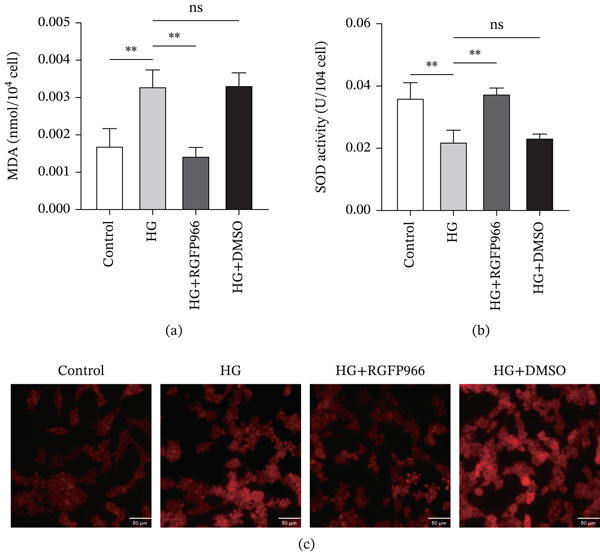
Inhibition of HDAC3 activity reduced the level of oxidative stress in 661W cells. (a) The MDA content of 661W cells in the control, HG, HG + RGFP966, and HG + DMSO groups. (b) The SOD activity of 661W cells in the control, HG, HG + RGFP966, and HG + DMSO groups. (c) The fluorescence intensity of ROS in 661W cells in control, HG, HG + RGFP966, and HG + DMSO groups. *n* = 3, ns, not significant, ∗*p* < 0.05, ∗∗*p* < 0.01, ∗∗∗*p* < 0.001, and ∗∗∗∗*p* < 0.0001. Scale = 50 *μ*m.

We divided the 661W cells into control group, HG group, HG + RGFP966 group, and HG + DMSO group. After cell adhesion, each group was treated accordingly for 48 h before subsequent assays. The MDA assay showed that MDA content was significantly elevated in the HG and HG + DMSO groups compared with the control group, whereas it was markedly reduced in the HG + RGFP966 group (Figure 9a). Conversely, SOD activity was significantly decreased in the HG and HG + DMSO groups but was increased in the HG+RGFP966 group (Figure 9b). ROS fluorescence staining further supported these findings: The HG and HG + DMSO groups exhibited stronger red fluorescence compared with the control group, indicating elevated intracellular ROS levels. In contrast, the HG + RGFP966 group showed notably weaker fluorescence intensity, suggesting that HDAC3 inhibition effectively suppressed high–glucose‐induced ROS accumulation (Figure 9c). These results indicate that RGFP966‐mediated HDAC3 inhibition mitigates oxidative stress in 661W cells under high glucose conditions.

### 3.8. HDAC3 Knockdown Attenuates Apoptosis in 661W Cells Under High Glucose Conditions

**Figure 10:**
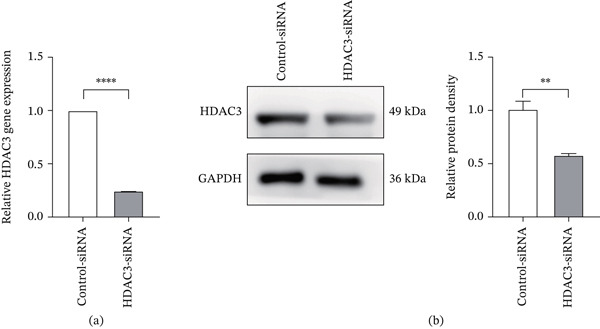
The expression level of HDAC3 in 661W cells was successfully downregulated by HDAC3‐siRNA. (a) Detection of the relative expression of HDAC3 mRNA in the 661W cells of the control‐siRNA and HDAC3‐siRNA groups by qPCR. (b) Detection of the relative protein expression of HDAC3 in the 661W cells of the control‐siRNA and HDAC3‐siRNA groups by western blotting. The internal reference is GAPDH, and the bar graph was drawn by ImageJ software to quantify the relative expression of HDAC3 protein in three independent experiments. *n* = 3, ns, not significant, ∗*p* < 0.05, ∗∗*p* < 0.01, ∗∗∗*p* < 0.001, and ∗∗∗∗*p* < 0.0001.

To further confirm the role of HDAC3, we knocked down HDAC3 expression in 661W cells using HDAC3‐specific siRNA. Cells were divided into control‐siRNA group and HDAC3‐siRNA group, and both were cultured under high glucose conditions. Successful knockdown of HDAC3 was verified at both the mRNA and protein levels by qRT‐PCR and western blot, respectively (Figure 10a,b).

**Figure 11:**
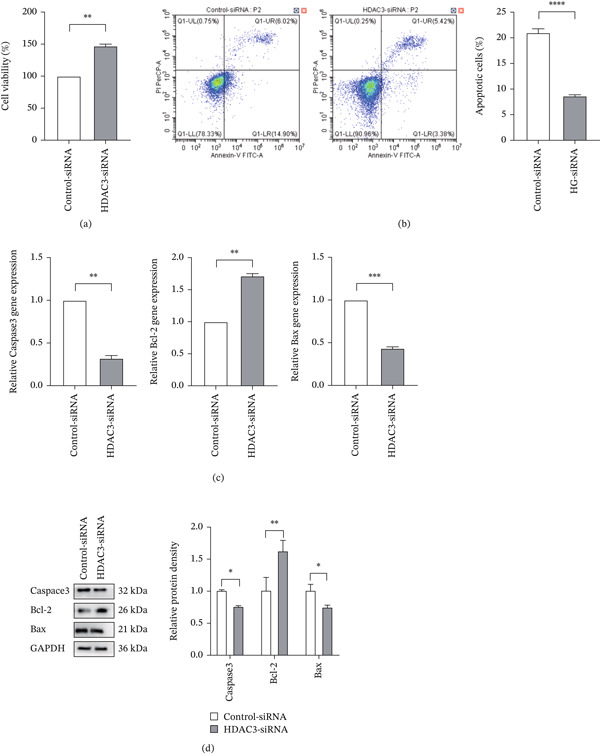
Knocking down the expression of HDAC3 reduced the level of apoptosis in 661W. (a) The cell viability of 661W cells under high glucose condition in the control‐siRNA and HDAC3‐siRNA groups detected by CCK8 assay. (b) Detection of the apoptosis level of 661W cells in the control‐siRNA and HDAC3‐siRNA groups by flow cytometry. The total apoptosis rate of each group was counted, and the bar chart was drawn. (c) Detection of the relative expression of Caspase3, Bcl‐2, and Bax mRNA in the 661W cells of the control‐siRNA and HDAC3‐siRNA groups by qPCR. (d) Detection of the relative protein expression of Caspase3, Bcl‐2, and Bax in the 661W cells of the control‐siRNA and HDAC3‐siRNA groups by western blotting. The internal reference is GAPDH, and the bar graph was drawn by ImageJ software to quantify the relative expression of Caspase3, Bcl‐2, and Bax protein in three independent experiments. *n* = 3, ns, not significant, ∗*p* < 0.05, ∗∗*p* < 0.01, ∗∗∗*p* < 0.001, and ∗∗∗∗*p* < 0.0001.

CCK‐8 assay showed that cell viability was significantly higher in the HDAC3‐siRNA group than in the control‐siRNA group after 48 h of high glucose exposure (Figure 11a). Flow cytometry analysis showed that the total apoptosis rate, as well as early and late apoptosis rates, was significantly lower in the HDAC3‐siRNA group compared with the control‐siRNA group (Figure 11b).

The results of qRT‐PCR assay indicated that HDAC3 knockdown led to a significant downregulation of Caspase‐3 and Bax mRNA expression, along with a marked upregulation of Bcl‐2 mRNA (Figure 11c). Western blot results further confirmed these trends at the protein level (Figure 11d). These results collectively demonstrate that genetic knockdown of HDAC3 effectively alleviates high–glucose‐induced apoptosis in 661W cells, further supporting the important role of HDAC3 in promoting photoreceptor injury under diabetic conditions.

### 3.9. HDAC3 Knockdown Attenuates Oxidative Stress in 661W Cells Under High Glucose Conditions

**Figure 12:**
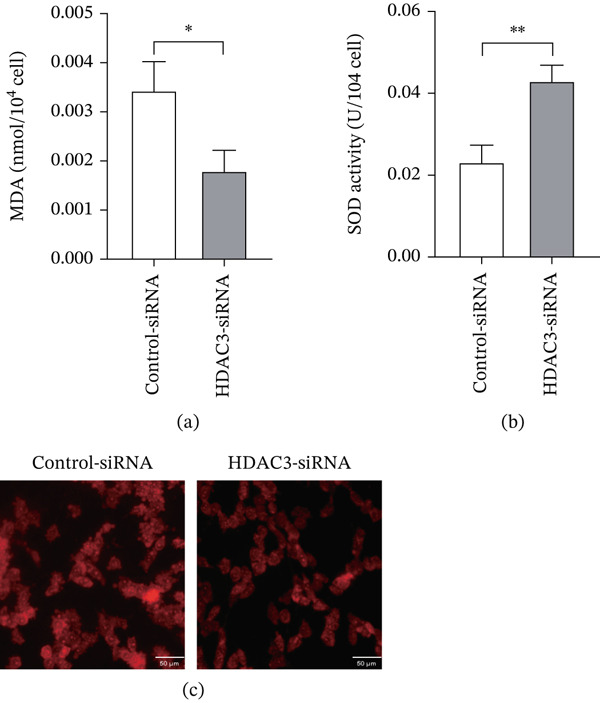
Knocking down the expression of HDAC3 reduced the level of oxidative stress in 661W. (a) The MDA content of 661W cells in the control‐siRNA and HDAC3‐siRNA groups. (b) The SOD activity of 661W cells in the control‐siRNA and HDAC3‐siRNA groups. (c) The fluorescence intensity of ROS in 661W cells in the control‐siRNA and HDAC3‐siRNA groups. *n* = 3, ns, not significant, ∗*p* < 0.05, ∗∗*p* < 0.01, ∗∗∗*p* < 0.001, and ∗∗∗∗*p* < 0.0001. Scale = 50 *μ*m.

To further validate the involvement of HDAC3 in high–glucose‐induced oxidative stress, 661W cells were transfected with control‐siRNA or HDAC3‐siRNA and cultured under high glucose conditions. MDA content was significantly lower in the HDAC3‐siRNA group compared with the control‐siRNA group (Figure 12a). Conversely, SOD activity was markedly increased following HDAC3 knockdown (Figure 12b). ROS fluorescence staining also demonstrated a clear reduction in red fluorescence intensity in the HDAC3‐siRNA group, indicating decreased intracellular ROS levels relative to the control group (Figure 12c). These results confirm that knockdown of HDAC3 effectively attenuates oxidative stress in 661W cells exposed to high glucose.

## 4. Discussion

DR remains one of the most common complications of diabetes, characterized by a highly complex and multifactorial pathogenesis that has yet to be fully elucidated. In the past few years, research efforts have primarily centered on vascular abnormalities in DR. However, growing evidence now underscores the critical contribution of retinal neurodegeneration to disease progression. A large number of studies have shown that oxidative stress and cell apoptosis play a crucial role in the pathogenesis and progression of DR, being core events of retinal neurodegeneration and vascular dysfunction, and are closely associated with the development and progression of the disease [31]. One study established a diabetic rat model via STZ induction and observed retinal tissue by electron microscopy. Results showed sporadic apoptosis of photoreceptor cells as early as 4 weeks after successful modeling, with pronounced apoptosis evident at 12 and 24 weeks. However, the underlying mechanisms remain unclear [32], and research focusing on photoreceptor pathology and its related molecular mechanisms in DR remains limited. In the present study, we established a diabetic mouse model and performed HE staining on retinal sections. We observed a significant decrease in the thickness of the entire retinal layer and the photoreceptor layer in diabetic mice at 8 and 12 weeks after diabetes induction, accompanied by structural disorganization. These results not only confirm the involvement of photoreceptors in DR progression but also emphasize the need to elucidate their specific role in the pathophysiology of the disease.

HDACs are a class of enzymes that mediate epigenetic modifications by regulating chromatin structure and gene expression [33]. Among them, HDAC3, which localizes to both the nucleus and cytoplasm, has been implicated in a wide range of physiological and pathological processes. Studies have shown that HDAC3 is associated with various diseases, including cancer, inflammation, metabolic diseases, and neurodegenerative disorders [34–36], by modulating key cellular processes such as cell proliferation, apoptosis, angiogenesis, metastasis, and antitumor resistance [37]. In recent years, the involvement of HDAC3 in diabetes and its complications has gained increasing attention. Elevated HDAC3 expression has been reported in models of DR [38, 39]. One animal study found elevated HDAC3 levels in the retinas of DR mice, along with increased oxidative stress, inflammation, and apoptosis. Treatment with the HDAC3‐specific inhibitor RGFP966 reduced HDAC3 expression and ameliorated oxidative stress, inflammation, and apoptosis in the retinas of DR mice. These findings suggest a close association between HDAC3 and the pathological processes of DR, and that inhibiting HDAC3 expression can alleviate DR‐related damage [40]. Lundh et al. demonstrated that numerous HDACs, particularly classical Class I HDACs, have been implicated in neurodegeneration. Lundh et al. demonstrated distinct regulatory roles for HDAC family members in cytokine‐induced apoptosis, revealing that Class I HDAC inhibitors can mitigate cytokine‐mediated damage, whereas Class II inhibitors lack this effect. Further investigation showed that only knockout of HDAC1 or HDAC3 within Class I HDACs successfully reduced apoptosis. Epigenomic analysis indicated that specifically HDAC3 gene knockout affected the binding of NF‐*κ*B to the iNOS promoter. These findings underscore HDAC3′s significant and somewhat unique role as a key regulator of apoptosis [41, 42]. Further research revealed that inhibiting HDAC3 effectively protected RGCs from damage. Subsequent experiments in live mice revealed that HDAC3 enables cells to initiate the intrinsic apoptotic program more rapidly upon injury. To address mechanistic questions difficult to study in vivo, the team utilized 661W cells as an in vitro model, discovering that HDAC3 triggers BAX/BAK activation and intrinsic apoptosis by activating the JNK signaling pathway and upregulating the p53 target gene PUMA. Their results provide valuable insights for our work [28]. These collective findings underscore the central role of HDAC3 in the pathogenesis of DR and support its potential as a therapeutic target.

The pathological mechanisms underlying DR are exceedingly complex and remain incompletely elucidated. Extensive research indicates that oxidative stress and apoptosis are central to the pathogenesis and progression of DR, constituting significantly to both retinal neurodegeneration and vascular dysfunction [31, 43, 44]. To further elucidate the relationship between HDAC3 and oxidative stress and apoptosis in photoreceptor cells under hyperglycemic conditions, we treated 661W cells with high glucose in an in vitro model. Our results revealed a significant upregulation of HDAC3 expression in 661W cells under high glucose conditions, aligning with in vivo findings from diabetic mice. Furthermore, a notable reduction in 661W cell viability was observed in the HG group, accompanied by increased rates of total apoptosis and the apoptosis rate in each stage. This was evidenced by the upregulation of apoptotic markers Caspase‐3 and Bax, and the downregulation of the antiapoptotic marker Bcl‐2. Additionally, we observed elevated levels of oxidative stress markers, including MDA and ROS, along with a decreased total activity of SOD. These in vitro findings suggest that HDAC3 expression is significantly upregulated in photoreceptor cells under high glucose conditions and is positively correlated with the degree of apoptosis and oxidative stress.

To further validate our hypothesis, we employed the HDAC3 inhibitor RGFP966 and utilized HDAC3‐siRNA to knock down HDAC3 expression in 661W cells. The results demonstrated that reducing the expression of HDAC3 could effectively significantly enhance cell viability under high glucose conditions. This intervention also markedly decreased both the overall apoptosis rate and stage‐specific apoptosis rates. Moreover, we observed a downregulation of apoptotic markers Caspase‐3 and Bax, coupled with an upregulation of the antiapoptotic marker Bcl‐2. Additionally, the levels of oxidative stress markers, MDA and ROS, were reduced, whereas the total activity of SOD increased. The above results indicate that diminishing HDAC3 expression mitigates high–glucose‐induced apoptosis and oxidative stress in photoreceptor cells. This evidence supports the notion that HDAC3 is a critical player in the pathological processes of DR in photoreceptor cells.

In conclusion, our results establish that HDAC3 was positively correlated with photoreceptor apoptosis and oxidative stress under high glucose conditions. Inhibition of HDAC3 expression could effectively mitigate the increase of apoptosis and oxidative stress induced by high glucose in 661W cells, suggesting that HDAC3 may be a potential therapeutic target for photoreceptor cell injury in DR. This provides a novel approach for early intervention and treatment of DR lesions.

However, our study has several limitations. Retinal pathology was examined only at selected time points in the diabetic mice, and the correlation between HDAC3 and oxidative stress/apoptosis–related factors was not verified by in vivo experiments. The protective effect of HDAC3 inhibition has also not yet been validated in vivo. Furthermore, the specific molecular mechanisms by which HDAC3 regulates apoptosis and oxidative stress require further investigation. In future studies, we will address these limitations by performing additional in vivo animal experiments involving HDAC3 knockdown and observing changes in oxidative stress markers such as MDA and SOD, thereby strengthening the connection between cellular and animal studies. This will be combined with more comprehensive time‐course analyses and in vivo models to elucidate the detailed pathways involved in HDAC3‐mediated cellular processes. HDAC3 is a widely distributed HDAC protein in cells. Inhibiting its expression has a protective effect on retinal nerve cell damage. We queried single‐cell RNA sequencing databases, which revealed that HDAC3 is expressed in multiple cell types within the human retina, including cone photoreceptor cells, rod photoreceptor cells, bipolar cells, horizontal cells, Müller glial cells, and RGCs. Our current experimental research is only focused on photoreceptor cells. In subsequent studies, some samples of DR can be collected to validate the specific localization of HDAC3 expression within retinal cell types.

## 5. Conclusions

Our study demonstrates that under high glucose conditions, HDAC3 expression was upregulated, and the levels of oxidative stress and apoptosis were increased in 661W cells. Inhibition or gene knockdown of HDAC3 effectively attenuated these pathological processes. These findings suggest that targeting HDAC3 may offer a promising therapeutic approach for mitigating photoreceptor cell injury in DR.

## Author Contributions

Conception and design: Songfu Feng and Lin Ling. Data collection: Qi Fang and Jiali Li. Data analysis and interpretation: Yulin Ma, Jiaqi Liu, and Qiuxia Lin.

Drafting the article: Qi Fang. Revising it critically for important intellectual content: Baoyi Liu. Qi Fang, Jiali Li, and Baoyi Liu contributed equally to this project and should be co‐first authors.

## Funding

This study was supported by President Foundation of ZhuJiang Hospital, Southern Medical University (yzjj2023ms19) and Basic and Applied Basic Research Project of Guangzhou Science and Technology Plan jointly the City and University (Nos. 202201020008 and 2023A03J0584).

## Disclosure

All authors read and approved the final manuscript.

## Ethics Statement

The study adhered to the tenets of the Declaration of Helsinki and was approved by the Medical Ethics Committee of Zhujiang Hospital of Southern Medical University (Reference Number: LAEC‐2023‐172).

.

## Conflicts of Interest

The authors declare no conflicts of interest.

## Data Availability

The datasets and models used and analyzed during the current study are available from the corresponding authors on reasonable request.
